# Dual-Task Interference: The Effects of Verbal Cognitive Tasks on Upright Postural Stability in Parkinson's Disease

**DOI:** 10.4061/2010/696492

**Published:** 2010-02-14

**Authors:** J. D. Holmes, M. E. Jenkins, A. M. Johnson, S. G. Adams, S. J. Spaulding

**Affiliations:** ^1^School of Occupational Therapy, The University of Western Ontario, London, ON, Canada N6G 1H1; ^2^Department of Clinical Neurological Sciences, Schulich School of Medicine and Dentistry, The University of Western Ontario, London, ON, Canada N6A 5A5; ^3^School of Health Studies, The University of Western Ontario, London, ON, Canada N6A 5B9; ^4^School of Communication Sciences and Disorders, The University of Western Ontario, London, ON, Canada N6G 1H1

## Abstract

Although dual-task interference has previously been demonstrated to have a significant effect on postural control among individuals with Parkinson's disease, the impact of speech complexity on postural control has not been demonstrated using quantitative biomechanical measures. The postural stability of twelve participants with idiopathic Parkinson's disease and twelve healthy age-matched controls was evaluated under three conditions: (1) without a secondary task, (2) performing a rote repetition task and (3) generating a monologue. Results suggested a significant effect of cognitive load on biomechanical parameters of postural stability. Although both groups increased their postural excursion, individuals with Parkinson's disease demonstrated significantly reduced excursion as compared with that of healthy age-matched controls. This suggests that participants with Parkinson's disease may be overconstraining their postural adjustments in order to focus attention on the cognitive tasks without losing their balance. Ironically, this overconstraint may place the participant at greater risk for a fall.

## 1. Introduction

Postural instability is a frequent and incapacitating symptom of Parkinson's disease (PD) and is only modestly responsive to pharmacotherapy [1]. As a result, patients must often resort to the use of attentional strategies such as mentally rehearsing action sequences, or consciously attending to their balance, to maintain equilibrium [2, 3]. The role of attention in PD has often been investigated within a dual-task paradigm; a methodology which requires participants to perform a primary task (e.g., postural control) while simultaneously performing a secondary task, which may be cognitive (e.g., speech) or motoric (e.g., carrying an object). 

Despite the considerable research that has evaluated the impact of dual-task interference on gait and upper extremity performance [4], few studies have examined the effects of dual-task performance on posture, among individuals with PD. 

Early work in this area employed clinically relevant measurements, rather than biomechanical assessments. Smithson et al. [3] evaluated standing balance in a sample of individuals with PD, both with and without the addition of a motoric secondary task (self-initiated movements, including arm raise, functional reach, and bend reach). Results suggested that postural instability was greatest among individuals with PD and also that this instability increased with the addition of a secondary task. Morris et al. [5] measured the effect of dual-task interference on postural stability, using a combination of motoric secondary tasks similar to those used by Smithson et al. [3] and a cognitive secondary task (reciting the days of the week backwards). Like Smithson et al. [3], Morris et al. [5] identified greater postural disturbances among individuals with PD and found that both motoric and cognitive secondary tasks produced a significant deterioration in performance, as compared with healthy age-matched controls. In both of these studies, a stopwatch was used to record the time to postural instability, defined as a change in stance position, or a demonstrable need for external support.

Later research employed a more rigorous biomechanical approach, in all cases utilizing a force platform to quantify postural instability. Secondary tasks included color judgment [6], sequential finger movement, and arithmetic calculation [7], and a visuospatial cognitive task [8]. In all three of these studies, secondary task interference produced significantly greater postural change among individuals with PD, as compared with healthy age-matched controls. 

Although these studies have contributed to our understanding of the effects of a secondary task on postural stability in people with PD, the impact of cognitively demanding tasks on postural stability is of sufficient interest to warrant further study. In this study, we will evaluate the following hypotheses:

1. individuals with PD will exhibit more dual-task interference than control participants;
2. that as the complexity of the secondary task increases, the effects of dual-task interference will be more pronounced on measures of postural instability.

## 2. Methods

### 2.1. Participants

Twelve participants with a clinical diagnosis of idiopathic Parkinson's disease (eight men), and 12 age-matched controls (eight men) participated in this study. Individuals with PD were recruited voluntarily from a movement disorders clinic in Southwestern Ontario. Control participants were recruited from within the PD community, and were (in some cases), care partners, family members, or friends of clinical participants.

Participants were between the ages of 50 and 80, and there were no significant age differences between groups (PD: M  = 64.00, SD  = 9.08; Control: M  = 62.67, SD  = 8.11). Participants were excluded from the study if they were experiencing any neurological (other than PD), cognitive, or motoric impairments that might impact on speech, mobility, or cognition. Additionally, individuals with a Modified Hoehn and Yahr score greater than 3 were excluded from the study, as these individuals have (by definition) difficulty standing without assistance, and were considered to present an unacceptable risk of falling. Participants with PD were evaluated on Section 3 of the Unified Parkinson Disease Rating Scale, and this clinical measure was undertaken by a neurologist specializing in movement disorders. All evaluations were done by the same individual. This clinical information is presented in Table 1, along with relevant demographic information.

### 2.2. Procedure

Participants with Parkinson's disease were tested during their self-determined peak, or “ON”, phase of their medication cycle. To help ensure that all participants were within their “ON” phase, testing was conducted approximately two hours after individuals took their usual medications, per the recommendations of Gauntlett-Gilbert and Brown [9]. Participants were asked to stand on the force platform, using a comfortable stance. This stance was then traced on a piece of clear plastic, and this tracing was used to reposition the feet for each subsequent trial. These tracings were then measured to assess stance length and width.

Each participant completed six 30-second trials on the force platform—two trials within each of three experimental conditions. Postural stability of each participant was evaluated with their eyes open under conditions of increasing complexity: (1) no secondary task; (2) while performing a numerical recitation task (counting from one to five in a looped sequence); (3) while engaging in a monologue (describing a familiar place). Trials were averaged within each condition for all data analyses that follow. To minimize the likelihood that participants would become familiar with the speech tasks, practice trials were prohibited. 

The research protocol, recruitment method, and mechanism for obtaining informed consent were approved by the Health Sciences Research Ethics Board, at the University of Western Ontario (review #11940 E).

### 2.3. Apparatus

Kinetic variables were collected using a model OR6-5 biomechanics platform (Advanced Mechanical Technology Inc., Watertown, USA), oriented so that the x-axis aligned in the direction of forward stance. Custom software was designed, using the sensitivity matrix (S_ij_) provided by the company. Accuracy was re-established at the outset of the experiment, by applying known weights in specific locations on the force platform. Calibration data were collected, and it was determined that the known input was matched by the output of the programs, thus ensuring true information was gathered using the force platform. Force components in the 3 principal axes (anterior-posterior, medial-lateral, and vertical) and moments about these axes were collected at 100 Hz [10].

### 2.4. Outcome Measures

The following outcome parameters were used as measures of postural stability.

1. Total length of the centre of pressure path in the horizontal plane on the force plate (COPL).
2. Maximal medial lateral COP excursion range, expressed as a percentage of the base of support (%BOSml), where base of support was defined as width of stance.
3. Maximal anterior posterior COP excursion range, expressed as a percentage of the base of support (%BOSap), where base of support was defined as length of stance.

**Figure 1:**
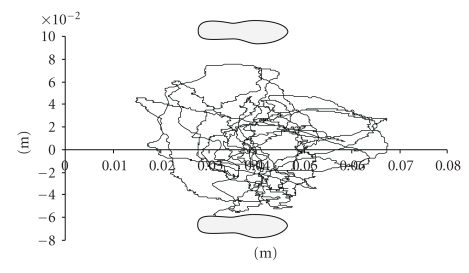
Schematic representation of postural variables.

A schematic representation of these variables is presented in Figure 1. 

### 2.5. Statistical Analysis

To evaluate the influence of dual-task performance on spontaneous centre of pressure excursion, a repeated measures split-plot analysis of variance was performed for each of the dependent variables (COPL, %BOSml, and %BOSap). For each of these analyses, the within-subject factor was “cognitive complexity” with 3 levels (Baseline, Numerical Recitation, and Monologue), and the between-subject factor was “participant group” with 2 levels. Reverse Helmert contrasts were used to elucidate significant effects of the interaction between group and task. This type of contrast allows for a post-hoc assessment of significant effects, by comparing each subsequent level with the average of the previous levels (i.e., “Low Complexity” is compared with “Baseline”, and “High Complexity” is compared with the average of “Baseline” and “Low Complexity”). In effect, this provides a means of evaluating the contribution of each increase in cognitive complexity within the secondary task. All statistical calculations are accompanied by estimates of effect size, calculated as an eta-square.

## 3. Results

**Figure 2:**
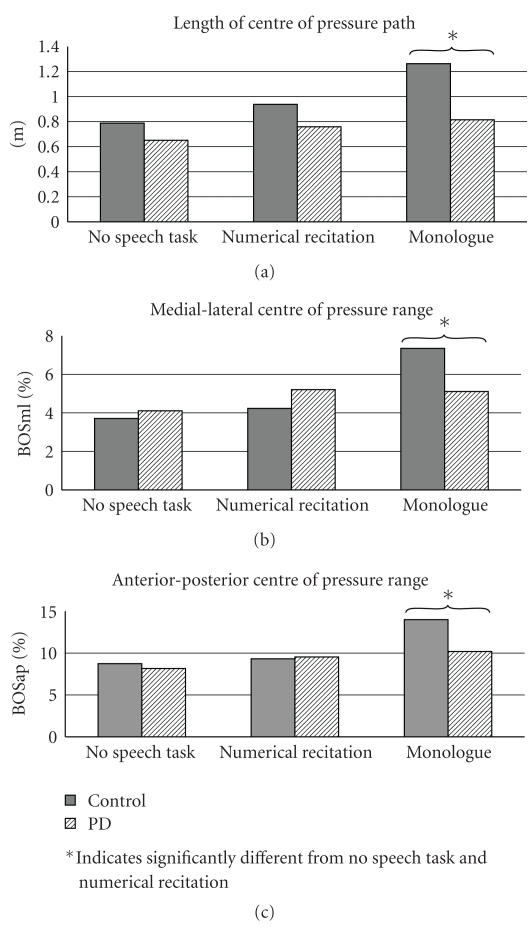
Graphical representation of group by task interaction, across all postural variables; *indicates significantly different from no speech task and numerical recitation see also Table 2.

The means and standard deviations for each dependent variable, separated by group, are presented in Table 2. A significant multivariate effect was found for the condition by group interaction (*F*(6,17) = 3.535, *P* = .019, *η* ^2^ = 0.55), suggesting that the interaction between condition and group predicts 55% of the variability in postural stability (as measured by an optimally weighted composite of the three dependent variables). Univariate testing suggested a statistically significant interaction between condition and group for all three dependent measures: COPL (*F*(2,44) = 15.518, *P* = .001, *η* ^2^ = 0.165); %BOSap (*F*(2,44) = 3.325, *P* = .045, *η* ^2^ = 0.0929); and %BOSml (*F*(2,44) = 5.228, *P* = .009, *η* ^2^ = 0.141). This suggests that the percentage of variance accounted for by the interaction between condition and group was 16.5% for COPL, 9.29% for %BOSap, and 14.1% for %BOSml. Evaluation of the reverse-Helmert contrasts suggested that only the most complex of the secondary cognitive tasks (i.e., conversational monologue) produced a significant amount of dual-task interference as compared with quiet stance and numerical recitation. These contrasts are summarized in Figure 2. Interestingly, individuals with Parkinson's disease showed significantly less excursion along both anterior-posterior and medial lateral axes, and demonstrated a significantly smaller centre-of-pressure pathway during the most complex task. These contrasts are also summarized in Figure 2.

## 4. Discussion

Consistent with previous research, this study demonstrates a significant effect of cognitive load on postural stability [7, 8, 11, 12]. In general, dual-task interference produced increased excursion of the centre of pressure, and this effect became more pronounced with increases in task complexity. Conversely, these data demonstrated a paradoxical effect among participants with Parkinson's disease. In keeping with previous research, tasks of low complexity result in an increased excursion of the centre of pressure, across all participants. This effect is, however, reversed on tasks of high complexity. With high complexity, participants with Parkinson's disease demonstrate reduced excursion, relative to the healthy age-matched controls. This effect is statistically significant along both axes (anterior-posterior and medial-lateral), and for the length of the centre of pressure pathway.

This suggests that participants with Parkinson's disease may be overconstraining their postural adjustments. Given that the overconstraint occurred under conditions of increased cognitive load, it is conceivable that individuals with Parkinson's disease were overconstraining their posture in order to focus attention on the cognitive tasks without losing their balance. Their posture was, therefore, stabilized beyond normal levels, in an attempt to prevent threats to balance that may occur when cortical resources are directed to the cognitive tasks. In other words, the patients “prepared themselves” prior to the performance of the cognitive task, by stabilizing to a greater extent than the healthy older controls. Ironically, this overconstraint (which may be undertaken by participants as a consequence of an elevated fear of falling) places the participant at greater risk for a fall.

Although studies of dual-task interference have not reported this overconstraining effect, previous research has demonstrated that individuals with Parkinson's disease have decreased excursion in the anterior-posterior during quiet stance, as compared with age-matched controls [13].

A “posture-first principle” has been proposed to account for changes in posture under dual-task conditions, wherein the individual copes with complex situations by prioritizing balance over other concurrent tasks [14]. In the current study, individuals with Parkinson's disease applied the “posture-first principle” to a pathological level by overconstraining their postural adjustments. Overconstraint results in reduced proprioceptive feedback, and may theoretically increase co-contraction, both of which diminish the individual's ability to respond to unexpected perturbations of balance. Consequently, this strategy for coping with dual-task interference places an individual with Parkinson's disease at a greater risk for falls when in a community setting.

## 5. Conclusions

These results suggest an important new direction for research within the dual-task paradigm. The maladaptive strategy implemented by individuals with Parkinson's disease may be addressed with meta-cognitive training that focuses on the development of a more appropriate posture-first strategy.

## Figures and Tables

**Table 1:** Clinical features of individuals with Parkinson's disease.

Subject	Gender	Age (y)	Duration of Illness (y)	Hoehn & Yahr	UPDRS III	Medication	Dosage total/day
PD1	Female	67	2	1.5	19	None	—
PD2	Male	78	8	2.5	36	Levodopa/Carbidopa	650 mg
						Pramipexole	2.25 mg
PD3	Male	69	3	2.0	23	Levodopa/Carbidopa	350 mg
PD4	Male	58	4	2.5	24	Pramipexole	1.5 mg
PD5	Female	50	9	2.5	20	Levodopa/Carbidopa	400 mg
						Ropinirole	9.5 mg
PD6	Male	60	2	1.5	17	Pramipexole	1.5 mg
PD7	Female	76	3	2.5	26	Levodopa/Carbidopa CR	400 mg
PD8	Male	59	5	2.0	26	Levodopa/Carbidopa	300 mg
						Pramipexole	3.0 mg
PD9	Male	49	4	2.0	28	Ropinirole	12.0 mg
PD10	Female	67	8	2.0	21	Levodopa/Carbidopa CR	200 mg
						Levodopa/Carbidopa	200 mg
PD11	Male	67	6	2.5	21	Levodopa/Carbidopa	1000 mg
						Pramipexole	2.0 mg
PD12	Male	68	5	2.0	28	Levodopa/Carbidopa	400 mg
						Pramipexole	0.75 mg
						Entacapone	800 mg
	Mean	64.00	4.92	2.13	24.08	—	—
	(SD)	(9.08)	(2.39)	(0.38)	(5.16)		

**Table 2:** Means (and standard deviations) for each postural variable, separated by group.

Healthy older adults			
	COPL (m)	BOSml (%)	BOSap (%)
No task	0.79 (0.11)	3.71 (1.32)	8.74 (2.55)
Numerical recitation	0.94 (0.15)	4.23 (1.52)	9.31 (3.11)
Monologue	1.26 (0.23)	7.35 (4.19)	14.0 (6.52)

Parkinson's disease			

	COPL (m)	BOSml (%)	BOSap (%)

No task	0.65 (0.11)	4.11 (2.57)	8.16 (2.07)
Numerical recitation	0.76 (0.16)	5.21 (3.87)	9.54 (3.36)
Monologue	0.82 (0.16)	5.11 (3.03)	10.20 (3.85)

Where COPL = length of centre of pressure path; BOSml = medial-lateral centre of pressure excursion range; BOSap = anterior-posterior centre of pressure excursion range.
